# An Outbreak of Aeromonas Hydrophila Food Poisoning in Deptsang Village, Samdrup Jongkhar, Bhutan, 2016

**Published:** 2016-12-24

**Authors:** Tsheten Tsheten, Dorji Tshering, Kinley Gyem, Sangay Dorji, Sonam Wangchuk, Tenzin Tenzin, Lungten Norbu, Tshering Jamtsho

**Affiliations:** ^a^ Royal Center for Disease Control, Ministry of Health, Bhutan; ^b^ Jomotsangkha Dungkhag, Samdrup/Jongkhar, Bhutan; ^c^ Minjiwoong Basic Health Unit, Jomotsangkha Dungkhag, Samdrup/Jongkhar, Bhutan

**Keywords:** Food Poisoning, *Aeromonas hydrophila*, Red Meat, Bhutan

## Abstract

**Background:** An outbreak investigation was carried out to determine the cause and confirm the source of food poisoning in Deptsang village for implementing prevention and control measures.

**Methods:** We conducted a retrospective cohort study for the outbreak investigation. Stool specimens were collected from cases to perform culture and antibiogram. The team also inspected the environment and hygiene practices in both the construction site and the entire community. The association between the exposure to carcass meat and their outcome of acute gastroenteritis was assessed by risk ratio. *P*<0.05 was considered statistically significant.

**Results:** Fifty-five villagers consumed the carcass meat during lunch and dinner resulting in 33 cases. Multi-drug resistant *Aeromonas hydrophila* was isolated from stool specimens of cases, which were susceptible to chloramphenicol only. A risk ratio of 2.1 was found between those people who consumed the carcass meat and those who did not consume the carcass meat (*P*<0.001).

**Conclusions:** The current outbreak of food poisoning was caused by the consumption of carcass meat contaminated with *A. hydrophila*. Provision of health education with emphasis on food hygiene is needed in remote areas to prevent such outbreaks in the future.

## Introduction

Foodborne diseases (FBDs) are a problem of global community entrenched mostly in developing countries. Although essential for survival, food or water can also be a vehicle of transmission of harmful microbes, which includes bacteria, viruses, parasites and chemicals or toxins. World Health Organization (WHO) estimates that billions of people are at risk of FBDs and millions fall ill every year^1^. While FBDs do not always have a lasting impact, they can affect productivity, wellbeing, healthcare expenditure, and in some cases, they have the potential to create chronic, lifelong health problems^2^. The symptoms of FBDs can vary from mild to self-limiting (diarrhea, nausea, and vomiting) to life-threatening conditions such as kidney and liver failure, disorder of nervous system, paralysis and cancers leading to long periods of absenteeism from work and premature death. A significant proportion of gastrointestinal illnesses are caused by foodborne pathogens^3^. To ensure food safety, rapid detection of these pathogenic organisms is very important^4^. One of the emerging bacterial pathogen associated with FBDs is *Aeromonas hydrophila*, frequently found in raw meat and drinking water. A greater risk of infection is reported in young children, elderly people and immunocompromised patients^5^.

On 25^th^ May 2016, District Health Officer (DHO) of Samdrup/Jongkhar district reported a cluster of acute gastroenteritis from Deptsang village to Royal Center for Disease Control (RCDC) through National Early Warning Alert and Response Surveillance (NEWARS) system. NEWARS is an ongoing systematic and adhoc collection of health-related data on selected diseases of public health importance and their associated syndromes from health centers in Bhutan^6^. The initial report hypothesized that the consumption of carcass meat was the likely source of the outbreak. On 22^nd^ May 2016, carcass meat was served to labor working in the construction of community temple during the lunch. The same meat was also consumed later at dinner by some of the villagers at their home. On recommendation by RCDC for outbreak investigation, the District Rapid Response Team (DRRT) was immediately formed to carry out the investigation.

The objective of the investigation was to determine the cause and confirm the source of the outbreak for implementing timely prevention and control measures.

## Methods

### Epidemiological investigation

Deptsang village has 33 households with 99 populations. At the time of the outbreak, villagers were busy with their labor contribution in the construction of community temple. Therefore, an additional three other people from another place also visited the village to assist before the outbreak in the construction of temple, which totaled to 102 populations at-risk. All these populations regardless of their age and sex were included in the analysis.

A retrospective cohort study was designed for the outbreak investigation. All 102 populations were retrospectively followed up from 22^nd^ to 27^th^ May 2016 for the development of any signs and symptoms of food poisoning after exposure to carcass meat. A case (ill) was defined as any person of any age living in Deptsang village, Samdrup/Jongkhar district that developed diarrhea with or without nausea, vomiting, abdominal cramps, headache or fever from 22^nd^ to 27^th^ May 2016. Non-case (not ill) included all another person who did not develop these signs and symptoms during the same period. Since the villagers had consumed regular food items besides carcass meat of cow, the information was collected only on exposure to carcass meat served on 22^nd^ May 2016 using a structured questionnaire from both cases and non-cases. The meat was believed to be rotten with foul smell even felt at several meters away. The information on when and how long the cow was dead was not ascertained. The owner of the deceased cow sought the help of her neighbor to carry the remains to the construction site as well as to their neighbors for the consumption.

### Environmental investigation

The kitchen that served the labors working in the temple construction was inspected for its hygiene and sanitation. The cook who prepared the meals for the labors was also examined physically for his hygiene and the way the foods was prepared.

### Laboratory investigation

Stool specimens were collected from 11 cases and tested in Basic Health Unit (BHU) at Jomotsangkha. This BHU provides health care services to the people living in Deptsang village. A stool wet mount was prepared using 0.85% normal saline and observed using a light microscope for cells, ova and parasites. Furthermore, the same specimens were transported to RCDC for laboratory testing and confirmation. Briefly, suspensions of stool specimens were made in 0.85% normal saline. The suspension was enriched in Buffered Peptone Water (BPW), Alkaline Peptone Water (APW) and Preston, as well as directly plated on Mac-Conkey Agar, Hecton Enteric Agar, and modified Charcoal Cefoperazone Deoxycholate Agar (mCCDA). All media were incubated aerobically except for mCCDA incubated at microaerophilic atmosphere at 37 ⁰C. The enriched suspension was also plated on all the above media and incubated at the same atmospheric condition. The suspected colonies from Mac-Conkey and Hecton Enteric Agar were identified as *Aeromonas* hydrophila biochemically using oxidase test, Kligler Iron Agar (KIA), indole, bile esculin, lysine decarboxylase (LD), ornithine decarboxylase (OD), arginine dihydrolase (AD) and also by using Biomerieux Analytical Profile Index (API) 20-E. Identified organism was then subjected to antimicrobial susceptibility testing using disc diffusion method as per the Clinical Laboratory Standard Institute (CLSI) guideline^7^. Test was performed on Mueller Hinton Agar plates using following antibiotic disks: amoxicillin (10 μg), cefazolin (30 μg), cephalexin (30 μg), ciprofloxacin (5 μg), ceftriaxone (30 μg), gentamycin (10 μg), nalidixic acid (30 μg), tetracycline (30 μg), sulfamethoxazole (25 μg) and chloramphenicol (30 μg).

## Statistical analysis

The demography of the cases was presented in terms of number, percentage, mean and standard deviation. Their distributions were presented graphically by using their date of onset of illness. The risk ratio was used to assess the association between the suspected foods (i.e., carcass meat) and the presenting illness. The categorical variables were analyzed by either Pearson Chi-Square or Fisher exact test depending upon the expected frequency, while continuous variable was analyzed by student’s *t*-test. The *P*<0.05 was considered statistically significant. Epi Info 7 (CDC, Atlanta, USA) was used for the analysis.

Ethical clearance was not required from Research Ethics Board of Health (REBH), Ministry of Health, Bhutan, in response to emergencies or outbreak investigation. The investigation was carried out to control and prevent further transmission of infection without incurring any risks or burden to the respondents.

## Epidemiological investigation

A combination of 55 villagers consumed the carcass meat during lunch and dinner resulting in 33 cases. The cases presented watery or loose diarrhea (100%), nausea/vomiting (67%), fever (40%), abdominal cramp (34%), giddiness (13%), disorientation (7%), jaundice (7%) and restless (7%). The majority of the cases were male with mean age of 38.1 yr (interquartile range = 28 yr). By occupation, farmers had the highest proportion of 66.7% followed by hermit and pre-school children with proportions of 14.7% and 10.8% respectively (Table 1).

**Table 1:** General characteristics of cases and non-cases during the food poisoning outbreak in Deptsang village, 2016

**Characteristics**	**Disease (n = 33)**		**Non disease (n = 69)**		***P*** ** value**
	**n**	**%**	**n**	**%**	
Gender					0.693
Male	20	57.8	39	56.5	
Female	13	42.2	30	43.5	
Occupation					0.001
Farmer	19	57.6	49	71.0	
Hermit	10	30.3	5	7.2	
Pre-school children	0	0.0	11	15.9	
Students	2	6.1	1	1.4	
Business	2	6.1	3	4.3	

**Figure 1:**
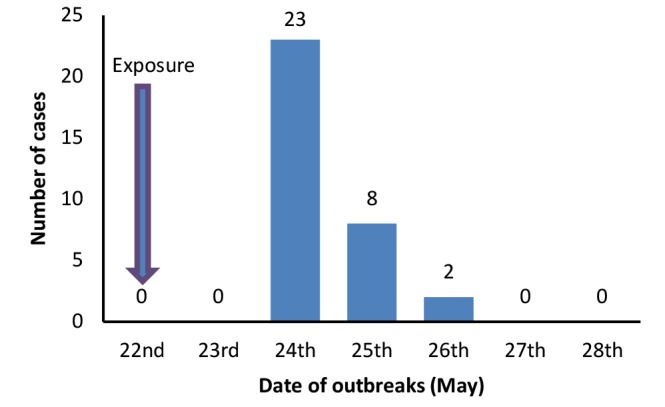
Epidemic curve of food poisoning outbreak in Deptsang village, 2016

The carcass meat was served both at the temple and at home on 22^nd^ May 2016. The cases were observed two days after exposure and lasted for three days from 24^th^ to 26^th^ May. The epidemic curve showed a typical common point source outbreak (Figure 1).

Twenty-nine people consumed the carcass meat at home and 26 people at the construction site. Among those consumed, 30 people got ill, while among those who did not consume, three of them were ill. A risk ratio of 2.1 was found between those who consumed the carcass meat and those who did not consume it. The association was found highly significant with *P*<0.001 (Table 2).

**Table 2:** Analysis of risk ratio between those who consumed carcass meat and those who did not consume it during food poisoning outbreak in Deptsang village, 2016

**Exposure status**	**Disease**	**Non-disease**	**Risk Ratio** **(95% CI)**	***P*** ** value**
Not exposed	3	44	1.00	
Exposed	30	25	2.10 (1.53, 2.78)	0.001

### Environmental investigation

The labors working in the construction site were served with foods prepared with firewood instead of electricity. The kitchen premise was found clean with a continuous water supply. The sole cook who managed to prepare all culinary items was also found neat and healthy. The hygiene and sanitation of every household were also found adequate with toilet and water tape connected to their houses. On further investigation, fecal matter of bear was observed around the area where the cow carcass was laid. The remaining of carcass intestine was also seen entangled with the plastic sack, for which the villagers' belief might have succumbed the cow.

### Laboratory investigation

Stool specimens tested positive for *Entamoeba histolytica* (2/11), *Entamoeba coli* (1/11), *Ancylostoma duodenale* (1/11) and *Taenia* species (1/11) in the saline mount. *Aeromonas hydrophilla* was isolated from a culture further confirmed by API 20E. White blood cells (WBCs) and red blood cells (RBCs) were sparingly seen in both watery and loose stools. The isolated organism was resistant to amoxicillin (5/5), cefazolin (5/5), cephalexin (5/5), ciprofloxacin (4/5), ceftriaxone (4/5), gentamycin (4/5), nalidixic acid (4/5), tetracycline (4/5) and sulfamethoxazole (4/5). All of the isolates (5/5) were susceptible to chloramphenicol.

## Discussion

The current outbreak seems to be associated with the carcass of cow contaminated with *A. hydrophila*. The distribution of cases by their onset over the period of two to four days after exposure to carcass meat was tantamount to the incubation period of *Aeromonas* species. Most of the cases (70%) had their onset after two days of exposure. Moreover, the findings of watery (secretory) and the non-dysenteric nature of stool among the cases hinges to *Aeromonas* as the cause of the current outbreak^8^. *Aeromonas* species are found in the intestine of animals and humans besides aquatic environment^9,10^. The intestines of carcass entangled with plastic sack were seen on the spot, which would have resulted from the infection of *Aeromonas*. A mixed infection of *E. histolytica*, *A. duodenale, Entamoeba coli* and *Taenia* species would indicate that they were exposed to other sources. It may not be related with the carcass of cow.

In the last decades, *Aeromonas* has been increasingly recognized as the etiological agent of gastrointestinal illness^11^. *Aeromonas* species are pathogens that cause foodborne gastroenteritis in humans with its virulence factors such as extracellular toxins (enterotoxins, hemolysin, protease, phospholipase, hydrolytic enzymes), structural features (pilli, S-layer, lipopolysaccharide), adhesion and invasion^12^. The outbreak has led to the hospitalization of 15 patients and referral of one patient to the regional hospital. The magnitude of the outbreak was augmented by the mixed infection of parasites in some cases. However, no casualty was reported with just one day of hospitalization among those admitted.

The susceptibility of the pathogen was observed to chloramphenicol only. The antibiotic resistant pattern of the pathogen is in agreement with a study conducted in South Africa and Iran particularly about amoxicillin^13,14^. However, those studies have shown high resistance to chloramphenicol, which was in discordant with our study. Such a high level of resistance could be caused by an increasing and indiscriminate use of antibiotics in the medicine, veterinary, and agricultural sectors. Studies conducted on antimicrobial susceptibility pattern of *Aeromonas* species isolated from meat and other environmental samples have found that the most of the organism was resistant to amoxicillin and tetracycline, and resistant to chloramphenicol was variable^15,16^.

The investigation was limited by the laboratory incapability to isolate and identify organism from the carcass samples. The limitation was also inherent by the lack of information on other risk factors because team exclusively collected risk factors for the carcass meat of cow only. Not all stool specimens were collected from every case due to the short period of investigation and insufficient human resources and laboratory supplies.

The carcass meat stored in the houses were recalled to a faraway place and burnt together. The remnants were disinfected with hypochloride solution and buried under ground. Health education on sanitation and food hygiene was given to the entire people living in a village.

## Conclusions

*A. hydrophila* has caused an outbreak of food poisoning in a remote village of Deptsang after consuming a carcass meat. Based on the investigation, health education should be designed to include food hygiene and implemented to address susceptible populations in remote villages of Bhutan.

## Acknowledgments

The team thanks all the respondents for providing candid responses and cooperation during the entire investigation.

## Conflict of interest statement

The authors declare no conflict of interest.

## Highlights

- This is the first documented outbreak of food poisoning caused by Aeromonas hydrophila.
- Poor food hygiene is a public health concern in remote villages of Bhutan.
- Food poisoning can be prevented by proper food hygiene and effective health education.
